# Phylogenies of Microcystin-Producing Cyanobacteria in the Lower Laurentian Great Lakes Suggest Extensive Genetic Connectivity

**DOI:** 10.1371/journal.pone.0106093

**Published:** 2014-09-10

**Authors:** Timothy W. Davis, Susan B. Watson, Mark J. Rozmarynowycz, Jan J. H. Ciborowski, Robert Michael McKay, George S. Bullerjahn

**Affiliations:** 1 Canadian Centre for Inland Waters, Environment Canada, Burlington, ON, Canada; 2 Department of Biological Sciences, Bowling Green State University, Bowling Green, Ohio, United States of America; 3 Department of Biological Sciences, University of Windsor, Windsor, Ontario, Canada; Royal Netherlands Institute of Sea Research (NIOZ), Netherlands

## Abstract

Lake St. Clair is the smallest lake in the Laurentian Great Lakes system. MODIS satellite imagery suggests that high algal biomass events have occurred annually along the southern shore during late summer. In this study, we evaluated these events and tested the hypothesis that summer bloom material derived from Lake St. Clair may enter Lake Erie via the Detroit River and represent an overlooked source of potentially toxic *Microcystis* biomass to the western basin of Lake Erie. We conducted a seasonally and spatially resolved study carried out in the summer of 2013. Our goals were to: 1) track the development of the 2013 summer south-east shore bloom 2) conduct a spatial survey to characterize the extent of toxicity, taxonomic diversity of the total phytoplankton population and the phylogenetic diversity of potential MC-producing cyanobacteria (*Microcystis*, *Planktothrix* and *Anabaena*) during a high biomass event, and 3) compare the strains of potential MC-producers in Lake St. Clair with strains from Lake Erie and Lake Ontario. Our results demonstrated a clear predominance of cyanobacteria during a late August bloom event, primarily dominated by *Microcystis*, which we traced along the Lake St. Clair coastline downstream to the Detroit River's outflow at Lake Erie. Microcystin levels exceeded the Province of Ontario Drinking Water Quality Standard (1.5 µg L^−1^) for safe drinking water at most sites, reaching up to five times this level in some areas. *Microcystis* was the predominant microcystin producer, and all toxic *Microcystis* strains found in Lake St. Clair were genetically similar to toxic *Microcystis* strains found in lakes Erie and Ontario. These findings suggest extensive genetic connectivity among the three systems.

## Introduction

Cyanobacterial harmful algal blooms (CHABs) occur worldwide and their increasing prevalence has been associated with severe ecological and economic impacts across the marine-freshwater continuum [1]–[8]. Many CHAB genera include species and strains that can produce toxins and other bioactive compounds that present a risk to the health of humans and other animals [9]. CHAB genera, including *Microcystis*, *Anabaena* and *Planktothrix* are well known to have microcystin-producing strains [10] and all have been found in the Laurentian (North American) lower Great Lakes.

The Laurentian Great Lakes are a vital global resource, containing approximately 18% of Earth's available surface freshwater [11]. Over the past several decades these systems have been subjected to many anthropogenic pressures such as the introduction of non-native species (e.g., dreissenid mussels and round gobies) and eutrophication. Anthropogenic nutrient loading has contributed to the shift in phytoplankton community composition in the lower Great Lakes (Erie and Ontario). Accordingly, much of the research over the past two decades has focussed on elucidating the factors that control the dynamics of phytoplankton communities, primarily on CHABs, in these two lakes. Explanations have been postulated to include changes in bottom-up controls such as nutrient availability and light [12]–[17], physical factors like wind strength [18] and top-down controls including pelagic [19] and benthic grazing [20]–[21]. Furthermore, differences and dynamics among the genetic strains of cyanobacteria within blooms have also been investigated through field and laboratory experiments. [22]–[32].

**Figure 1:**
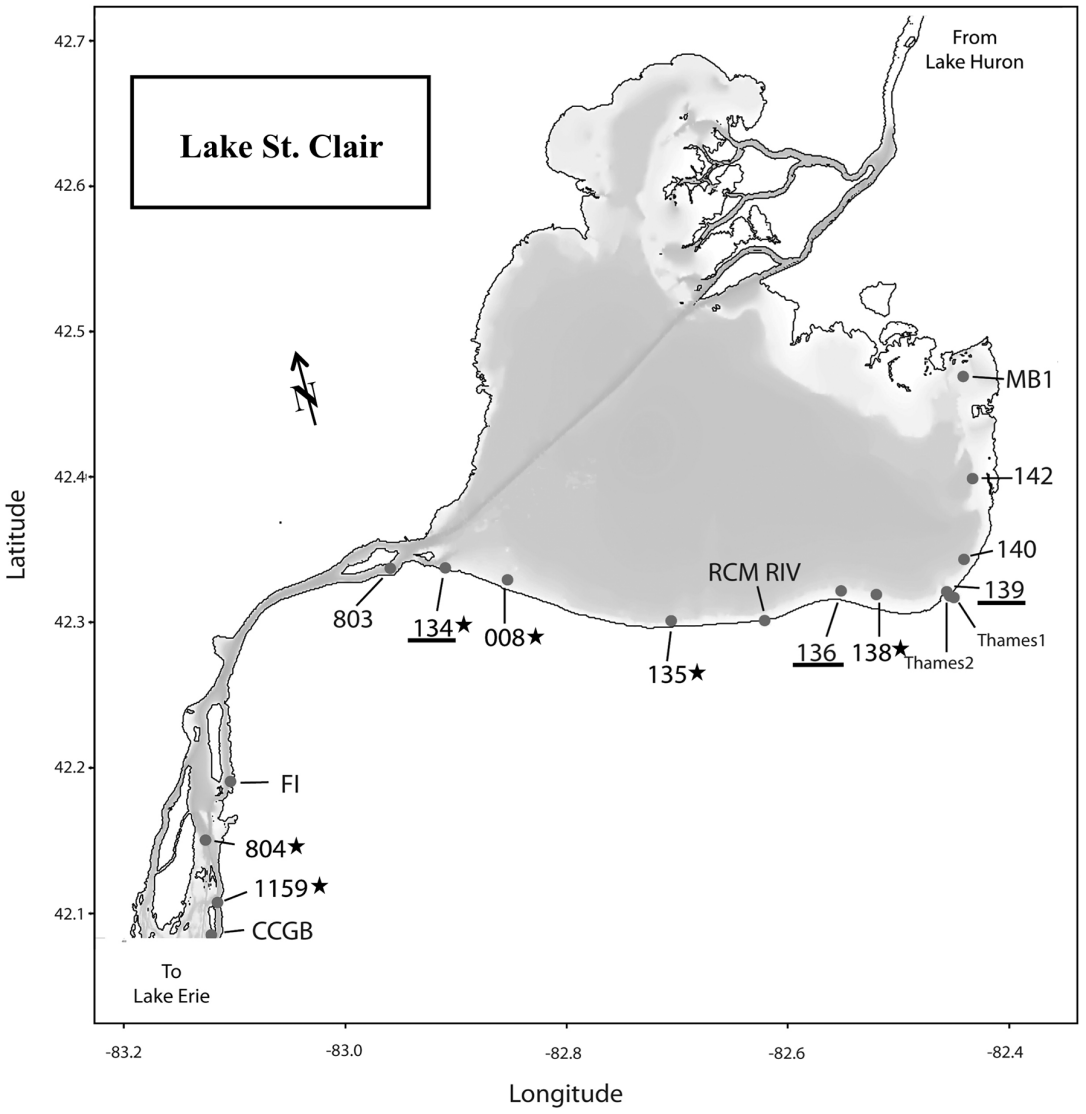
Map of Lake St. Clair indicating sampling sites. The underlined sites were the seasonal monitoring sites; starred sites indicate where DNA was extracted and sequenced for genetic diversity.

Lake St. Clair lies between Lake Huron and Lake Erie (Fig. 1). It receives water from Lake Huron via the St. Clair River and discharges to Lake Erie via the Detroit River, the largest tributary to Lake Erie [33]. Lake St. Clair also receives inflow from wastewater treatment plants and several tributaries, most notably the Thames River, which drains nearly 6,000 km^2^ of rich agricultural land in southwest Ontario. The Thames River flows into the southeast corner of the lake (Fig. 1) transporting elevated levels of nutrients to the inshore waters [34], [35]. To date, relatively few studies have focused on the planktonic component of the lower food web in Lake St. Clair. A few studies have documented the plankton ecology and community composition prior to the dreissenid mussel invasion [36]–[39]. Vijayavel et al. [40] recently documented the presence of the nuisance benthic cyanobacterium *Lyngbya wollei* for the first time along a recreational beach on the northwest shore of Lake St. Clair. However, despite anecdotal reports of blooms and MODIS satellite imagery suggesting that periods of increased biomass along the south-east near-shore waters occur during the summer months, no study has investigated the toxicity, taxonomic or molecular diversity of these blooms nor how they relate to the CHAB events observed in Lake Erie and even further downstream, in Lake Ontario. We hypothesized that Lake St. Clair may be an immediate source (i.e. days to weeks) of potentially toxic cyanobacterial biomass to the western basin of Lake Erie. Therefore, the goals of our study were to: 1) track the development of the 2013 summer south-east shore bloom 2) conduct a spatial survey to characterize the extent of toxicity, taxonomic diversity of the total phytoplankton community and the phylogenetic diversity of potential MC-producing cyanobacteria (*Microcystis*, *Planktothrix* and *Anabaena*) during a high biomass event, and 3) investigate the genetic connectivity of potential MC-producers in Lake St. Clair with strains from Lake Erie and Lake Ontario collected over the past 10 years, including two Great Lakes Areas of Concern in Lake Ontario that also experience CHAB events: Hamilton Harbour (43° 17′ 30.50″ N, 79° 49′ 45.02″ W) and the Bay of Quinte (44° 08′ 47.4″ N, 77° 15′ 51″ W).

## Methods

### Study sites

Lake St. Clair (42° 25′ 20″ N, 82° 39′ 36″ W) is a shallow (mean depth ∼3 m) waterbody with a surface area of 1100 km^2^ [41], [42] and is the smallest lake in the Laurentian Great Lakes system. Three Environment Canada sites (135, 136, 139; Fig. 1) along the south-east corner where the Thames Rivers discharges into Lake St. Clair were sampled from June through August 2013. Additionally, during a high biomass event on August 23^rd^, samples were collected at 17 locations along a more spatially resolved survey extending from Mitchell's Bay (Site MB1) to the outflow of Lake St. Clair at the mouth of the Detroit River (Site CCGB; Fig. 1). No provincial or federal permits or permissions were required to conduct this research as Lake St. Clair is a public waterbody and is not provincially nor federally protected.

### Sample collection

Water samples were collected bi-weekly at sites 134, 136 and 139 from a small, shore-launched boat (Fig. 1). Physicochemical data were measured at each site using a calibrated water quality probe (YSI, Yellow Springs, Ohio, USA). The parameters measured were surface water temperature, dissolved oxygen concentration, pH, and conductivity. Water samples were collected using a Van Dorn sampler from a depth of 1 m and kept on ice until returned to the lab for processing within four hours. From each site, subsamples were preserved in Lugol's iodine solution (1% final conc.) for phytoplankton cell identification and biovolume calculation, or filtered to collect material for cell-bound DNA analysis through a 0.22 µm Sterivex filter cartridge (Millipore Corp., Billerica, MA, USA) until no more water could pass through. The filter cartridges were immediately frozen and stored at −80°C until analysis.

A more extensive sampling protocol was employed for the spatial survey. The water quality data and the integrated water samples were collected at each site as described above. In addition, duplicate water samples for the analysis of dissolved nutrient (nitrate/nitrite [NO_3_^−^+NO_2_^−^], ammonia [NH_3_], soluble reactive phosphorus [SRP], dissolved total Kjeldahl nitrogen [DTKN] and total dissolved phosphorus [TDP] samples were collected by filtering lake water through a 0.45 µm×47 mm polycarbonate filter into triple rinsed 20 mL plastic bottles and stored at −20°C until analysis. Water samples for total Kjeldahl nitrogen [TKN] and total phosphorus [TP] analysis were collected by filling a triple rinsed 20 mL plastic vial with whole lake water followed by storage at −20°C. Before analysis, TP samples were thawed and preserved with 1% (v/v) H_2_SO_4_ then analyzed following persulfate digestion. All nutrient samples were analyzed at the National Laboratory for Environmental Testing in Burlington, Ontario using standard methods [43]. Particulate P [PP] values were calculated using the equation: [PP = TP – TDP]. Samples for total MCs were collected by pipetting 1 mL of whole lake water into a low-binding polycarbonate centrifuge tube and stored at -80°C until analysis. No protected or endangered species were sampled during any of these surveys.

### Phytoplankton identification and biomass determination

Samples were enumerated using the Utermöhl technique for algal biomass and taxonomic composition [44], [45]. Depending on sample density, subsamples of 2–5 mL were settled over 24 hours and counted at 100× or 400× using a Leica DM inverted phase microscope, enumerating a minimum of 100 settling units for the most abundant taxa. Colonies and filaments were measured individually and converted to cells using a regression estimate of average cells per unit biovolume [46]. Cell counts were converted to biomass (carbon) from average measured cell volumes and taxa were identified to genus level according to major taxonomic sources [47]–[58].

For this study, we restricted our classification of the phytoplankton community to broad taxonomic groups as a detailed taxonomic description of the overall phytoplankton community will be reported elsewhere (S. Watson et al., *in prep*). Furthermore, there is debate over the validity of the traditional *Microcystis* morphospecies classification with evidence indicating they are too genetically similar to be considered separate species [59]. However, other studies have indicated that this conclusion is premature until more is known about the drivers of the physiological and morphological diversity of this genus [53]. Therefore, for this study, we limited our identification of potential MC-producing cyanobacteria to the genus level.

### Extraction and analysis of microcystins

Total MCs were extracted from samples using a combination of physical and chemical lysis techniques. All samples were subjected to three freeze/thaw cycles before the addition of the QuikLyse reagents (Abraxis LLC; Warminster, PA, USA) as per the manufacturer's instructions. The samples were centrifuged for five minutes at 2 ×10^3^×*g* to pellet cellular debris. The concentrations were measured using an enhanced sensitivity microcystin enzyme-linked immunosorbent assay (Abraxis LLC; Warminster, PA, USA) following the methodologies of Fischer et al. [60]. This assay is congener-independent as it is sensitive to the ADDA moiety, which is found in almost all microcystins. These analyses yielded a detection limit of 0.04 µg L^−1^.

### DNA extraction and sequencing

DNA was extracted from the 0.22 µm Sterivex cartridges from six sites spanning the southern shoreline of Lake St. Clair (Fig. 1) and from Hamilton Harbour and the Bay of Quinte, Lake Ontario using the PowerWater Sterivex DNA Isolation Kit (MO BIO Laboratories, Carlsbad, CA, USA) according to the manufacturer's instructions. DNA concentration and purity was measured using a NanoDrop lite spectrophotometer (Fisher Scientific Inc., Ottawa, ON, Canada). 260/280 ratios between 1.8 and 2.0 were considered to be acceptable for PCR. All PCR amplifications were performed using *mcyA* primers that detect potential microcystin-producing genotypes in *Microcystis*, *Planktothrix* and *Anabaena* [61] and have been used in previous Great Lakes CHAB phylogenetic studies [23], [24], [29]. PCR conditions were similar to those described in Hisbergues et al. [61]. Briefly, an initial denaturation at 95°C for 10 min; 40 cycles of 94°C for 30 s, 59°C for 30 s, 72°C for 30 s, and a final extension step at 72°C for 5 min were performed. Amplified PCR products were separated using a 1% (wt./vol.) agarose gel and visualized using ethidium bromide. Samples presenting bands around 300 bp in length were selected for TOPO cloning using fresh PCR products.

A *mcyA* clone library was generated from the amplified PCR products by insertion into pCR4-*TOPO* TA vector (TOPO TA cloning kit Invitrogen/Life Technologies, Burlington, ON, Canada) and transformed into chemically competent One Shot TOP10 *Escherichia coli* cells. DNA sequencing was performed (Genewiz Inc., South Plainfield, NJ, USA) and the resulting sequences were trimmed and dereplicated using custom PERL scripts. Sequence alignment and phylogeny was completed using Mega 5.2 [62]. For a succinct comparison with previous studies, *mcyA* sequences generated in this study were clustered at 99% identity using UCLUST [63]; the most abundant sequence in the cluster was then used as the reference sequence for phylogenetic comparison. To compare the reference sequences from this study with *mcyA* sequences from previous studies in Lake Erie [23] and Lake Ontario [24], a Maximum-likelihood tree was generated using the Jones-Taylor-Thornton (JTT) algorithm [64] and bootstrap values were obtained for 1,000 replicates.

#### Nucleotide sequence accession numbers

Sequences were deposited in GenBank (accession numbers KJ418279 through KJ418338).

## Results

### Physicochemical parameters

Station depths at all three monitoring sites were between 1–2 m on all sampling dates. Water temperatures at each site were similar for each date, ranging from 16°C to 26°C (Table S1) and were within the temperature range for cyanobacterial growth [8], [ 65]–[69]. Similarly, both dissolved oxygen concentration and pH were fairly consistent among sites on each date but conductivity varied (Table S1).

For the spatially extended sample series, mean water depth (±SE) was 3.3±0.9 m and water temperature, conductivity and pH were consistent between sites (Table S2). Inorganic nitrogen (nitrate/nitrite & ammonia) concentrations fluctuated with the highest concentrations around the river mouth sites (*t*-test, p<0.05; Table S2). Soluble reactive phosphorus concentrations were generally low and were below detection limit at five sites (Table S2). DTKN and TDP were fairly consistent across all sites (Table S2). TP concentrations varied across the sampling sites, with higher concentrations being observed at the river mouth sites (Table S2). Finally, no correlations were found between concentrations of any nutrient and either *Microcystis* biomass or microcystin concentration during the spatial sampling survey (principal component analysis; data not shown). However, any conclusion based on these data must be tempered by the fact that they are limited to contemporaneous sampling of nutrients and phytoplankton on a single date.

### Phytoplankton community biomass and composition

**Figure 2:**
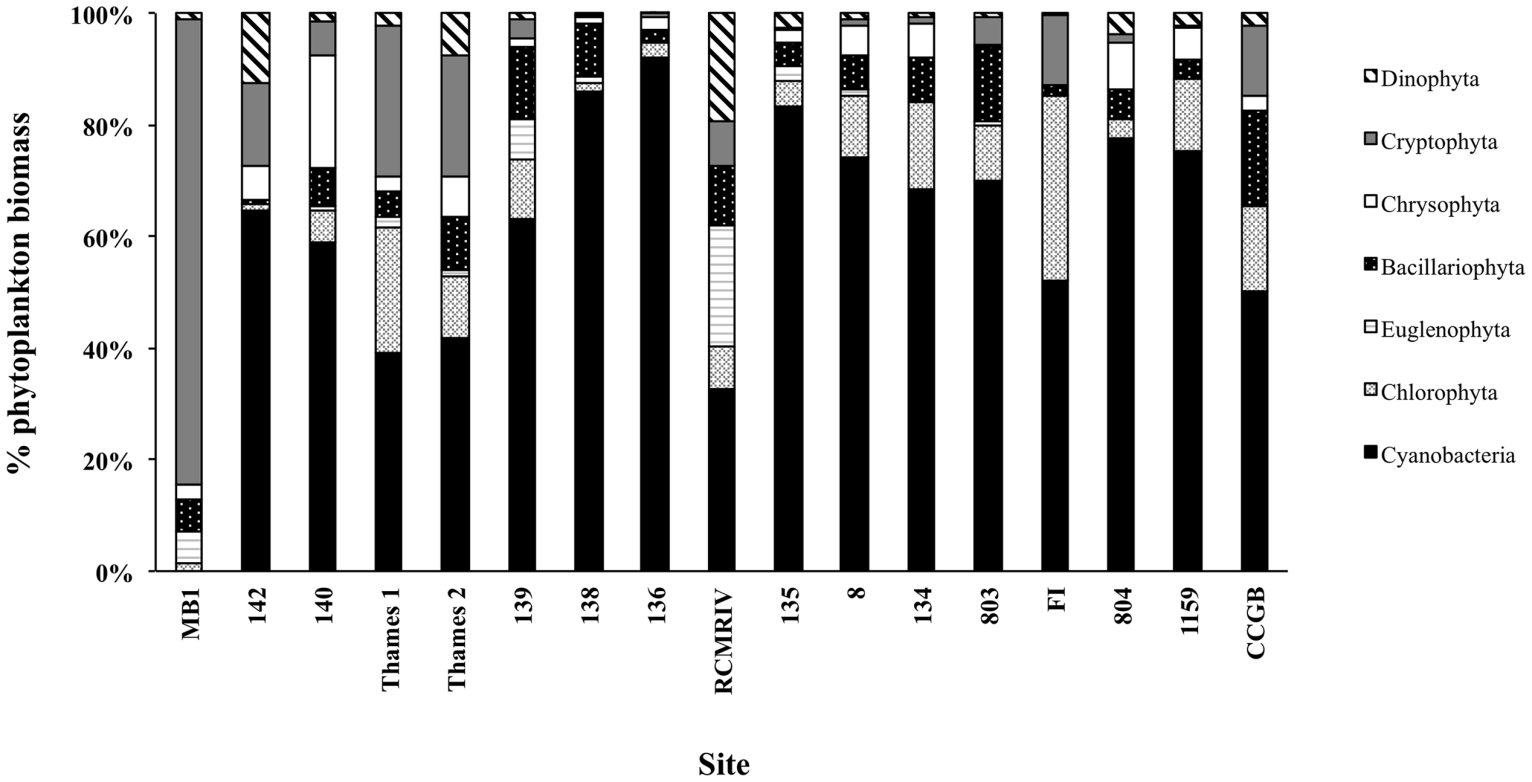
Percent biomass composition of the total phytoplankton community of the seven major phyla found in Lake St. Clair during the 23 August survey.

**Figure 3:**
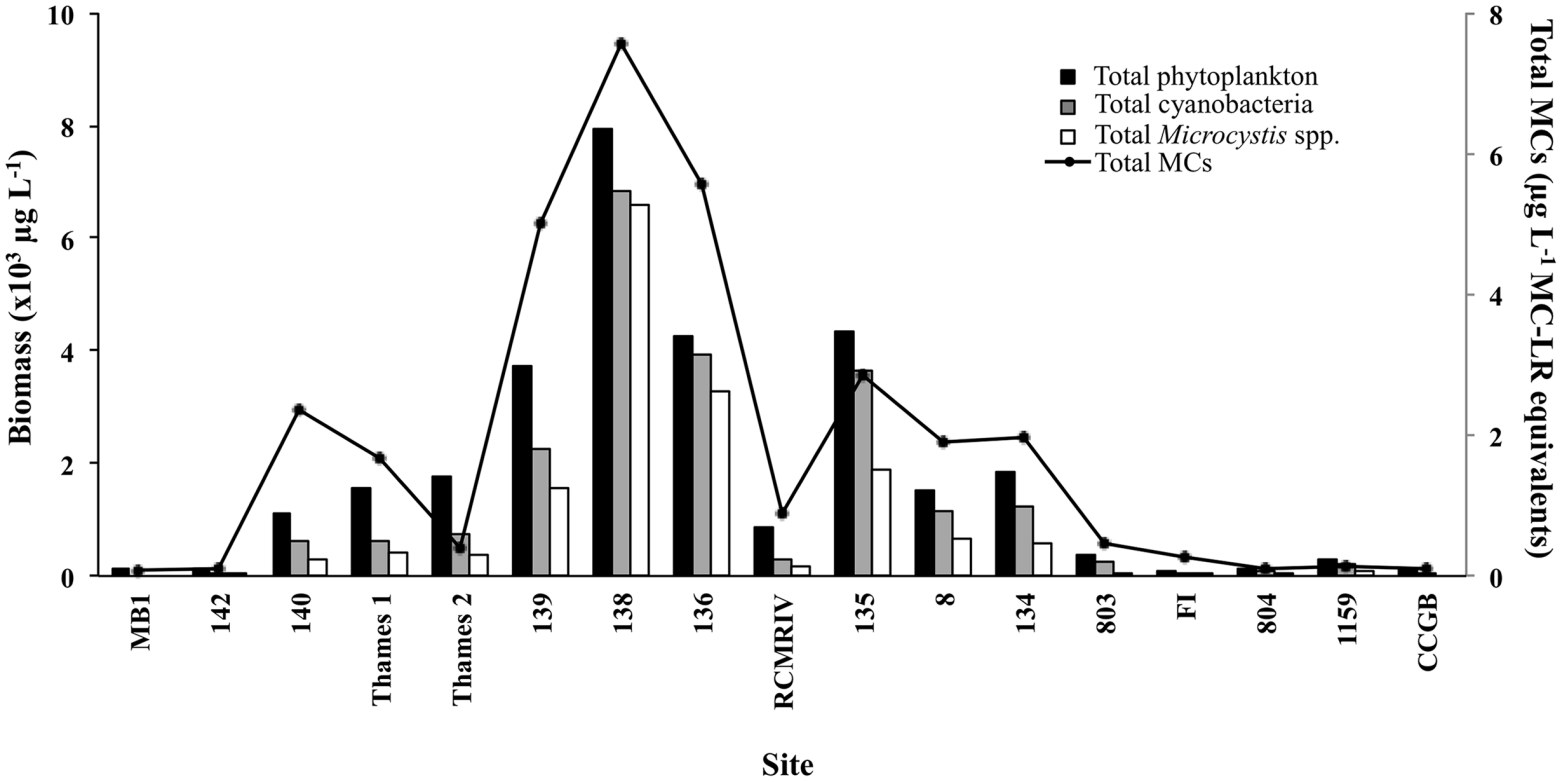
Total phytoplankton (black bars), cyanobacteria (grey bars) and *Microcystis* biomass (white bars) and total microcystins (MCs; solid black line) values at each site for the 23 August 2013 survey.

Over the entire sampling period, *Microcystis* biomass varied from below detection to 4.7×10^3^ µg L^−1^ at stations 134, 136 and 139 (Table 1). *Microcystis* biomass peaked at sites 134 and 136 in early August (Table 1) whereas at site 139 *Microcystis* biomass was either below detection or very low until late August when the survey was conducted (Table 1). Genera from all the major freshwater phytoplankton phyla were represented across the survey sites (Fig. 2). Total phytoplankton biomass ranged from 0.075×10^3^ to 7.9×10^3^ µg L^−1^ (Fig. 3). Cyanobacterial biomass ranged from below detection (site MB1) to 6.8×10^3^ µg L^−1^ (Figs. 2 & 3). Despite the fact that Lake St. Clair is generally considered as representative of Lake Huron water (i.e. low in nutrients and productivity), at 70% (12 of 17) of the sites sampled, cyanobacterial biomass comprised >50% of the overall phytoplankton biomass (Figs. 2 & 3), averaging 59±6% of the total biomass across all 17 sampling sites. Cryptophytes and chlorophytes were the next two dominant phyla comprising, on average, 12±5% and 10±2% of the total phytoplankton biomass (Fig. 2). All other phyla combined comprised <20% of the overall phytoplankton biomass (Fig. 2). For the 17 survey sites, total *Microcystis* biomass ranged from below detection limit (sites MB1 and CCGB) to 6.6×10^3^ µg L^−1^ at station 138 (Fig. 3). *Microcystis* was the only known potential-MC producer observed within the cyanobacterial community comprising >40% of the cyanobacterial biomass at 81% (13/16) of sites (Fig. 3).

**Table 1:** Total *Microcystis* biomass from the monitoring sites in Lake St. Clair during the field season of 2013.

Total *Microcystis* biomass (µg L^−1^)			
Date	Site 134	Site 136	Site 139
6-Jun	BDL	BDL	BDL
17-Jun	BDL	BDL	BDL
4-Jul	59	439	49
19-Jul	1849	1332	BDL
3-Aug	2202	4703	BDL
23-Aug	597	3047	1618

BDL =  below detection limit.

### Phylogenetic diversity of potential MC producers and bloom toxicity

**Figure 4:**
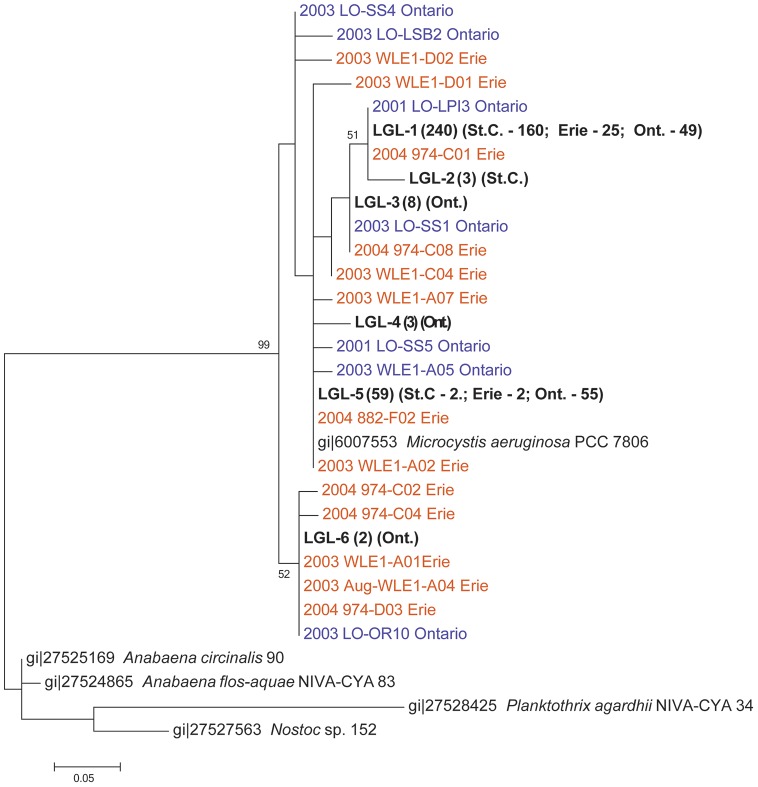
Maximum-likelihood tree of *mcyA* sequences sequenced from our study (LGL-1-6; bolded) and *mcyA* sequences from previous studies in Lake Erie (orange) and Lake Ontario (purple). Numbers in parentheses indicate the number of identical sequences represented by the named sequence or LGL group. St.C  =  Lake St. Clair; Ont.  =  Lake Ontario; Erie  =  Lake Erie. Bootstrap values of >50% (for 1,000 replicates) are displayed at the branch nodes. The scale bar represents substitutions per site.

Based on sequenced *mcyA* amplicons, phylogenetic assessment of six sites across the southern shore of Lake St. Clair through the Detroit River (Fig. 1) was consistent with microscopic analysis, and also pointed to the single genus, *Microcystis*, as the primary source of MC production during the 23 August bloom event (Fig. 4). Toxin concentrations ranged from 0.08 to 7.56 µg L^−1^, with peak concentrations occurring at site closest to the mouth of the Thames River (138; Fig. 3). Furthermore, MC concentrations were strongly correlated with total *Microcystis* biomass (ρ = 0.91, p<0.001; Spearman's correlation matrix) during the 23 August spatial survey.

Interestingly, there was little diversity between *mcyA* amplicons collected from each site in Lake St. Clair, with all of the amplicons clustering with previously reported *Microcystis aeruginosa mcyA* sequences (Fig. 4). Amplicons from the western basin of Lake Erie and throughout Lake Ontario were also included in the phylogenetic analysis. Clustering of the *mcyA* amplicons sequenced during this study from Lake St. Clair, Lake Erie and Lake Ontario at 99% identity revealed six groups; LGL-1 through LGL-6 (Fig. 4). Five of the six groups clustered together and were comprised of strains from all three systems (Fig. 4). LGL-6, which contained only strains from Lake Ontario formed a separate cluster but grouped with strains collected during previous studies in Lake Erie and Lake Ontario (Fig. 4). The clustering of strains from Lake St. Clair with strains from the two lower Great Lakes, suggests genetic connectivity of MC producers throughout these lower Great Lakes (Fig. 4).

## Discussion

This study is the first to investigate the spatial molecular and taxonomic diversity and toxicity of cyanobacterial blooms along the south shore of Lake St. Clair and the Detroit River. At 8 of the 17 survey sites (47%) the MC concentrations exceeded both the 1 µg L^−1^ guideline level for safe drinking water set by the World Health Organization as well as the Province of Ontario Drinking Water Quality Standard (1.5 µg L^−1^), and therefore could pose a risk to human health. Similarly to near shore regions of the two lower lakes (Erie and Ontario), our data show that the southern shore of Lake St. Clair has undergone a phytoplankton community shift, possibly due to increased nutrient loading and potential influence from invasive species (e.g. dreissenid mussels). Indeed, prior to the establishment of dreissenid mussel populations in Lake St. Clair, cyanobacterial biomass tended to be very low, even during late August at near shore sites that roughly aligned with our sites 139, 142, 135, 134 [37]–[39]. At that time, diatoms primarily dominated the phytoplankton community, with chrysophytes and cryptophytes present at most sites and chlorophytes present at fewer sites [37]–[39]. Our results suggest that the composition of the late summer (August-September) phytoplankton community has changed significantly and is now dominated by cyanobacteria, with *Microcystis* dominating the cyanobacterial community at most sites along the southern coast. Although our results reflect conditions in only a portion of Lake St. Clair and describe broad taxonomic groups; a detailed study of the specific species composition of the offshore and near shore phytoplankton community is forthcoming (S. Watson, unpublished data).

This is also the first study to investigate if toxic populations of *Microcystis* from Lake St. Clair may influence the bloom populations in the western basin of Lake Erie. Current belief is that blooms in the western basin of Lake Erie are seeded internally and derived from overwintering *Microcystis* cells [39]. However, much of the focus has been on the potential for toxic strains to enter Lake Erie via the Maumee River as it is a significant source of sediment and nutrients to the western basin [25], [37], [70], [71]. The potential contribution of toxic *Microcystis* strains from Lake St. Clair via the Detroit River has not been previously considered.

Importantly, our results show *Microcystis* strains at the mouth of the Detroit River that are genetically similar to strains in Lake St. Clair, strongly suggesting that Lake St. Clair is an active source of toxic *Microcystis* strains to the western basin of Lake Erie. It takes, on average, 19 hours for a parcel of water to travel down the Detroit River to Lake Erie (via the Amherstburg channel) [72]. Furthermore, even though *Microcystis* biomass was below detection limit, using traditional light microscopy, at the mouth of the Detroit River (site CCGB), this does not mean *Microcystis* cells were completely absent from the water column. Genetic analysis supports this claim as positive *mcyA* sequences that clustered with *Microcystis* spp. were obtained at site 1159, which is just upstream of the Detroit River mouth (Fig. 1). The Detroit River discharges into the western basin of Lake Erie at an average rate of 5800 m^3^ s^−1^ and accounts for approximately 90% of the hydraulic load [33]. It is feasible that the high *Microcystis* biomass near the head of the Detroit River is diluted due to the high flow rate and therefore below quantifiable limits in individual samples (e.g. 1–10 colonies L^−1^); nevertheless due to the sheer volume of water discharged from the Detroit River (5.8×10^6^ liters s^−1^; or >20 billion liters hr^−1^) it is reasonable to conclude that the total loading of biomass entering Lake Erie from Lake St. Clair via the Detroit River is sufficient to impact the toxic *Microcystis* populations in the western basin.

The clustering of Lake St. Clair strains with strains collected throughout Lake Erie and Lake Ontario from previous years suggests a genetic connectivity among the three lakes. Dyble et al. [25] found similar results in a comparison of *mcyB* sequences from Saginaw Bay, Lake Huron and the western basin of Lake Erie. They also found similar sequences between the two water bodies, unfortunately, the sequences reported in Dyble et al. [25] were generated using a different gene (*mcyB*) in the MC gene operon and could not be included in our evaluation. Our study relied on the *mcyA* gene for which there is a robust record of sequence data from both Lakes Erie and Ontario [23], [24], [29]. However, a bloom occurring in Saginaw Bay, Lake Huron is unlikely to have any short-term impact on western basin strain dynamics or toxicity due to the distance between Saginaw Bay and Lake Erie and the potential for Saginaw Bay waters to be significantly diluted by Lake Huron water. Based on these data, it is plausible that the genetic connectivity observed in our study extends into the upper Great Lakes (Michigan, Huron and Superior). However, further research needs to be conducted to fully investigate this.

Source-tracking and diversity of potential MC producers to the western basin of Lake Erie have been the focus of several previous studies [22], [23], [29]. Kutovaya et al. [29] investigated the postulate that the Maumee River, (Ohio, USA) may be a source of toxic *Microcystis* into the western basin of Lake Erie. With a watershed in excess of 16,000 km^2^ draining predominantly agricultural lands in the U.S. Midwest, the Maumee River is a significant source of sediment and nutrients to Lake Erie's western basin [15], [29], [70], [71], [73], [74], but its role in seeding toxic *Microcystis* to the lake was unclear. Results from that study indicated that *mcyA* sequences identified from the Maumee River were distinct from *mcyA* sequences isolated from the open waters of the western basin. Kutovaya et al. [29] concluded that *Planktothrix* spp. were primarily responsible for MC production in the river whereas *Microcystis* spp. were the primary MC producers in the western basin. Therefore, the Maumee River was an unlikely source of toxic *Microcystis* strains, although some doubt surrounds these conclusions. In contrast, our findings strongly suggest a link between the toxic *Microcystis* strains in Lake St. Clair and lakes Erie and Ontario. Clearly, the ecology of the blooms in Lake St. Clair must be studied in further detail to better understand how continued changes in water quality will impact the toxicity, density and duration of these toxic *Microcystis* blooms.

Furthermore, our results indicated a broad connectivity among populations of toxic *Microcystis* strains in Lake St. Clair, Lake Erie and Lake Ontario. Previous studies have investigated the genetic diversity of MC producing phytoplankton within Lake Erie [23] and within Lake Ontario [24] using the *mcyA* gene. We were able to incorporate those sequences into our analysis along with data from strains we have isolated from Hamilton Harbour and the Bay of Quinte. Both of the previous studies found *Microcystis* to be the primary MC producer in the main basin of the lake, similar to our results for Lake St. Clair. Both studies also found genetic differences in populations of *mcyA*-containing *Microcystis* collected from different parts of the system. We found that most *mcyA* sequences clustered together with only a small group, LGL-6, forming a separate cluster with strains from Lakes Erie and Ontario (Fig. 4). This could be due to conditions in Lake St. Clair during the bloom, which may have favoured one particular genotype of toxic *Microcystis* on the survey date. Clearly, this requires further investigation and Lake St. Clair should be sampled at other times to evaluate the genetic diversity toxic *Microcystis* community throughout the growing season (May – October). Although our findings suggest *Microcystis* is the primary MC producer in Lake St. Clair, other MC producers may occur at other times of the bloom season. Nonetheless, during this particular sampling period, *mcyA* fragments from *Microcystis* were preferentially amplified due to the dominance of *Microcystis* at this time and these sequences showed genetic homogeneity.

We also showed that the total *Microcystis* biomass was positively correlated with MC concentrations in Lake St. Clair. However, it has been well documented in many temperate lakes that over the course of a growing season, MC concentrations do not correlate with total *Microcystis* biomass. Rather, the shifts between subpopulations of toxic and non-toxic *Microcystis* strains within a bloom largely control the overall toxicity [75]–[76]. Therefore, we cannot extrapolate our findings over the entire growing season as the environmental conditions during our spatial survey may have been promoting toxic strains to dominate the *Microcystis* population leading to the observed correlation between biomass and MC concentration, which may not persist over time.

Overall, our study provides the first evidence that blooms along the south shore of Lake St. Clair are toxic and that MC concentrations reach levels that may pose a threat to human health. Furthermore we demonstrated a clear genetic connectivity between the lower Great Lakes indicating that Lake St. Clair is a potentially important immediate source of toxic *Microcystis* strains contributing to the Lake Erie western basin blooms. As we cannot address the possibility of the historical influence of toxic strains of *Microcystis* from the upper Great Lakes (e.g. Saginaw Bay) into Lake St. Clair and Lake Erie, future phylogenetic work using the universal *mcyA* marker should be conducted to determine if this genetic connectivity extends into Lake Huron and possibly into lakes Superior and Michigan. Future studies in Lake St. Clair must focus on understanding the environmental drivers (e.g. nutrients, light, temperature) of these toxic strains. More intense and earlier-forming blooms in Lake St. Clair could further influence the toxicity of blooms in the western basin of Lake Erie. Furthermore, mechanistic experiments need to be conducted in all three systems where *Microcystis* blooms tend to occur to elucidate any common environmental drivers. As we observed similarities in the genetic populations of the MC-producing communities in all three lakes, common factors are likely responsible for causing elevated toxicity in each system.

## Supporting Information

Table S1 **Physicochemical data from the three monitoring sites in Lake St. Clair during the field season of 2013.** BDL =  below detection limit.
(DOCX)

Table S2 **Physicochemical data from the survey sites on 23 August 2013.** Average values are for all sites sampled. SRP =  soluble reactive phosphorus; DTKN =  dissolved total kjeldahl nitrogen; TDP =  total dissolved phosphorus.
(DOCX)
