# Supplementary material for: Phylogenies of Microcystin-Producing Cyanobacteria in the Lower Laurentian Great Lakes Suggest Extensive Genetic Connectivity
Source: PLoS One. 2014 Sep 10;9(9):e106093. doi: 10.1371/journal.pone.0106093 (PMC4160157; doi:10.1371/journal.pone.0106093)
Supplement: Table S1 — Physicochemical data from the three monitoring sites in Lake St. Clair during the field season of 2013. BDL = below detection limit. (DOCX) [file pone.0106093.s001.docx]

| **Site** | **Date** | **Temperature (°C)** | **Dissolved oxygen (mg L^-1^)** | **Conductivity (S cm^-1^)** | **pH** |
| --- | --- | --- | --- | --- | --- |
| 134 | 6-Jun-13 | 16.1 | 10.9 | 195 | 8.2 |
|  | 17-Jun-13 | 21.9 | 8.4 | 280 | 8.4 |
|  | 4-Jul-13 | 22.8 | 9.3 | 261 | 8.6 |
|  | 19-Jul-13 | 26.2 | 10.1 | N/R | 8.9 |
|  | 3-Aug-13 | 22.3 | 8.1 | 226 | 8.8 |
|  | 23-Aug-13 | 24.5 | 8.8 | 241 | 8.5 |
| 136 | 6-Jun-13 | 17.6 | 9.4 | 417 | 8.3 |
|  | 17-Jun-13 | 22.6 | 7.6 | 377 | 8.5 |
|  | 4-Jul-13 | 23.1 | 9.5 | 540 | 8.4 |
|  | 19-Jul-13 | 25.7 | 7.7 | N/R | 8.8 |
|  | 3-Aug-13 | 21.8 | 8.1 | 249 | 8.9 |
|  | 23-Aug-13 | 23.9 | 8.1 | 254 | 8.7 |
| 139 | 6-Jun-13 | 19.4 | 8 | 571 | 7.9 |
|  | 17-Jun-13 | 21.4 | 6.3 | 585 | 7.6 |
|  | 4-Jul-13 | 22.6 | 6.9 | 530 | 7.7 |
|  | 19-Jul-13 | 24.9 | 6 | N/R | 8 |
|  | 3-Aug-13 | 22.7 | 5.8 | 615 | 7.9 |
|  | 23-Aug-13 | 23.1 | 8 | 370 | 8.6 |

Supplementary Table S1:
