# Supplementary material for: Phylogenies of Microcystin-Producing Cyanobacteria in the Lower Laurentian Great Lakes Suggest Extensive Genetic Connectivity
Source: PLoS One. 2014 Sep 10;9(9):e106093. doi: 10.1371/journal.pone.0106093 (PMC4160157; doi:10.1371/journal.pone.0106093)
Supplement: Table S2 — Physicochemical data from the survey sites on 23 August 2013. Average values are for all sites sampled. SRP = soluble reactive phosphorus; DTKN = dissolved total kjeldahl nitrogen; TDP = total dissolved phosphorus. (DOCX) [file pone.0106093.s002.docx]

| **Site** | **Temperature (°C)** | **Depth (m)** | **Dissolved oxygen (mg L^-1^)** | **Conductivity (S cm^-1^)** | **pH** | **NO_3_/NO_2_ (µg L^-1^)** | **NH_3_ (µg L^-1^)** | **SRP (µg L^-1^)** | **DTKN (µg L^-1^)** | **TDP (µg L^-1^)** | **TP (µg L^-1^)** |
| --- | --- | --- | --- | --- | --- | --- | --- | --- | --- | --- | --- |
| **MB1** | 23.4 | 1.8 | 8.63 | 217 | 8.89 | 28 | 20 | 0.2 | 245 | 3.2 | 10.6 |
| **142** | 23.6 | 1.0 | 8.05 | 195 | 9.09 | 17 | 17 | BDL | - | 1.2 | 6.6 |
| **140** | 23.8 | 2.9 | 8.44 | 257 | 8.87 | 312 | 19 | 0.2 | 168 | 4 | 20.6 |
| **139** | 23.1 | 4.5 | 8.04 | 370 | 8.55 | 784 | 28 | 0.3 | 287 | 6.7 | 48.1 |
| **T1** | 24.4 | 1.0 | 7.60 | 467 | 8.23 | 1310 | 44 | 0.4 | 320 | 23.9 | 50.1 |
| **T2** | 24.6 | 5.2 | 7.90 | 501 | 8.13 | 1510 | 53 | 1.1 | 585 | 12.3 | 55 |
| **138** | 24.1 | 1.4 | 7.38 | 260 | 8.73 | 278 | 27 | 3.1 | 297 | 3.7 | 32.4 |
| **136** | 23.9 | 0.9 | 8.13 | 254 | 8.70 | 296 | 21 | 1.4 | 282 | 9.7 | 33.4 |
| **RCM RIV** | 25.1 | 2.1 | 4.85 | 378 | 8.20 | 18 | 50 | 4.1 | 574 | 10.9 | 89.2 |
| **135** | 24.5 | 1.9 | 7.68 | 247 | 8.70 | 298 | 21 | 0.2 | 276 | 3.8 | 21 |
| **008** | 24.3 | 2.0 | 7.65 | 219 | 8.23 | 145 | 16 | 0.4 | 121 | 15.5 | 24.5 |
| **134** | 24.5 | 0.5 | 8.82 | 241 | 8.54 | 229 | 13 | 0.4 | 257 | 16 | 18.6 |
| **803** | 24.0 | 13.3 | 7.73 | 214 | 8.46 | 156 | 14 | BDL | 228 | 1.5 | 10.3 |
| **FI** | 24.2 | 0.5 | 7.76 | 218 | 9.26 | 9 | 17 | 0.5 | 288 | 3.4 | 11.4 |
| **804** | 23.7 | 9.7 | 7.37 | 219 | 8.90 | 251 | 26 | BDL | 229 | 1.4 | 6.7 |
| **1159** | 24.2 | 6.9 | 8.45 | 233 | 8.70 | 214 | 24 | BDL | 179 | 1.8 | 10.8 |
| **CCGB** | 24.5 | 1.3 | 9.35 | 215 | 8.87 | 229 | 16 | BDL | 194 | 2.8 | 6.9 |
| **Average (± SE)** | 24.1 (0.12) | 3.3 (0.87) | 7.9 (0.22) | 277 (23) | 8.70 (0.08) | 358 (106) | 25 (3.0) | 1.0 (0.37) | 267 (34) | 7.2 (1.6) | 27 (5.5) |

Supplementary Table S2
